# LIGHTing tumor up for checkpoint blockade

**DOI:** 10.18632/oncotarget.10531

**Published:** 2016-07-11

**Authors:** Yong Liang, Hua Peng

**Affiliations:** Key Laboratory of Infection and Immunity, Institute of Biophysics, Chinese Academy of Sciences, Beijing, China

**Keywords:** immune checkpoint blockade, PD-L1, tumor infiltrating lymphocytes, LIGHT, immunotherapy

Immune checkpoint blockade, as a breakthrough in cancer therapy with anti-PD-L1, anti-PD-1, and anti- CTLA4, has demonstrated impressive therapeutic effects in multiple clinical trials. However, only a small minority of patients respond to such therapies [1]. Higher tumor infiltrating lymphocytes (TILs) and PD-L1 expression in tumors have been found to correlate with better prognoses [2, 3]. However, due to a lack of proper tumor models, it is difficult to determine the exact contributions of PD- L1 expression or TILs to checkpoint blockade therapies. In a recent study published in *Cancer Cell*, Tang H. *et al.* performed an extensive investigation to address these issues, and provided strong evidence for designing therapeutic strategies to extend the benefits of checkpoint blockade for more patients [4].

The authors first examined the correlation between PD-L1 expression and the responsiveness to PD-L1 blockade in a number of mouse tumor models, which elicited very interesting results, as observed in clinical patients. Specifically, MC38 tumors responded well to PD-L1 blockade, while Ag104Ld tumors didn't respond to the same treatment at all. Further analysis showed that both of the tumors expressed similar high levels of PD- L1, indicating that PD-L1 expression is not sufficient to determine the therapeutic effect of PD-L1 blockade. Interestingly, a significantly greater number of TILs were found in MC38 tumors. Furthermore, therapeutic effects of PD-L1 blockade in MC38 tumors were completely abolished when lymphocyte infiltration was blocked. The critical role of TILs in tumor control by PD-L1 blockade was further confirmed in several other PD-L1 expressing tumor models.

It would be interesting to know whether promoting lymphocyte-infiltration into a tumor can increase the therapeutic effect of anti-PD-L1. Previous studies in the same group have shown that ectopic expression of LIGHT, a cytokine belonging to the tumor necrosis factor superfamily, is able to recruit and activate T cells in tumor tissues [5]. By interacting with lymphotoxin β receptors, LIGHT induces the production of various chemokines and adhesion molecules, which recruit immune cells. LIGHT can also provide co-stimulatory signals to T cells by binding to another receptor, herpesvirus entry mediator. However, the application of LIGHT for tumor immunotherapy in mouse models is limited due to the instability of the recombinant mouse-LIGHT protein. To overcome this issue, a mutated version of human LIGHT (hmLIGHT) was created, which can bind to both human and mouse receptors. Tumor-specific delivery of hmLIGHT has shown impressive anti-tumor effects in several different mouse models [4]. Unfortunately, the anti-tumor effects of hmLIGHT gradually reduced as the tumor progressed, and PD-L1 was found upregulated after treatment. In fact, intratumoral PD-L1 upregulation, which is usually induced by interferons released by TILs, has been found to be an adaptive immune-resistant mechanism to confront effector T cell functions [1]. The authors showed that additional PD-L1 blockade following LIGHT treatment completely eradicated large established tumors, while PD-L1 blockade or LIGHT alone failed in tumor control. Impressively, in tumors resistant to PD- L1 due to a lack of TILs, LIGHT treatment can restore their responsiveness by recruiting T cells into tumor tissues. Thus, this study demonstrates that sufficient lymphocyte infiltration into the tumor is a prerequisite for checkpoint blockade immunotherapy. LIGHT, through recruiting lymphocytes, is able to reshape the tumor microenvironment and restore the responsiveness to checkpoint blockade in non-T cell-inflamed tumors.

It is a challenging but rewarding proposal to develop targeting-cytokines that can increase T cell infiltration for tumor control, due to their potential side effects. In this case, fusing LIGHT to a tumor targeting antibody solved the problem for delivering sufficient cytokines into the tumor while avoiding systemic toxicity. In another recent study, employing RGR peptides to specifically deliver LIGHT into the tumor environment also demonstrated impressive modulating effects, even with a physiological treatment dose [6]. In the future, more carrier molecules could be applied to specifically target different tumors for cytokine delivery.

As proof-of-concept, combining PD-L1 blockade with LIGHT provides a new strategy to expand its anti-tumor effects to a broader range of tumors. Since the presence of spontaneous TILs correlates with better prognoses in many immunotherapies, it would be interesting to test whether other immunotherapies such as anti-CTLA4 or IDO inhibitors could be further improved by LIGHT. Also, manipulating the tumor microenvironment through LIGHT in combination with traditional small-molecule drugs, tumor-associated neoangiogenic inhibitors, or tumor metabolism modulators may also provide distinct advantages that regulate the host immune response, cellular trafficking, and infiltration to the tumor microenvironment [7].
